# Coverage, determinants of use and repurposing of long-lasting insecticidal nets two years after a mass distribution in Lihir Islands, Papua New Guinea: a cross-sectional study

**DOI:** 10.1186/s12936-021-03867-z

**Published:** 2021-08-04

**Authors:** Pere Millat-Martínez, Rebecca Gabong, Núria Balanza, Sakaia Luana, Sergi Sanz, Silvia Raulo, Arthur Elizah, Chilaka Wali, Benjamin Paivu, Julian Dalmas, Samson Tabie, Stephan Karl, Moses Laman, William Pomat, Oriol Mitjà, Bàrbara Baro, Quique Bassat

**Affiliations:** 1 ISGlobal, Hospital Clínic—Universitat de Barcelona, Barcelona, Spain; 2 Lihir Malaria Elimination Programme (LMEP), Lihir Island, Papua New Guinea; 3 Consorcio de Investigación Biomédica en Red de Epidemiología y Salud Pública (CIBERESP), Madrid, Spain; 4 Department of Basic Clinical Practice, Faculty of Medicine, Universitat de Barcelona, Barcelona, Spain; 5 Australian Institute of Tropical Health and Medicine, James Cook University, Smithfield, Australia; 6 Papua New Guinea Institute of Medical Research, Goroka/Madang, Papua New Guinea; 7 Fight AIDS and Infectious Diseases Foundation, Badalona, Spain; 8 Infectious Disease Department, Hospital Universitari Germans Trias i Pujol, Badalona, Spain; 9 Department of Clinical Sciences, Faculty of Medicine and Health Sciences, University of Barcelona, Barcelona, Spain; 10 Lihir Medical Centre, International SOS, Lihir Island, Papua New Guinea; 11 ICREA, Pg. Lluís Companys 23, 08010 Barcelona, Spain; 12 Pediatrics Department, Hospital Sant Joan de Déu, Universitat de Barcelona, Esplugues, Barcelona, Spain; 13 Centro de Investigação em Saúde de Manhiça (CISM), Maputo, Mozambique

**Keywords:** Bed net, Coverage, Long-lasting insecticidal net, LLIN, Malaria, Papua New Guinea, Repurposing, Vector control

## Abstract

**Background:**

Universal coverage with long-lasting insecticidal nets (LLINs) is an essential component of malaria control programmes. Three-yearly mass distribution of LLINs in Papua New Guinea (PNG) has been successful in reducing infection transmission since 2009, but malaria prevalence ramped up from 2015 onwards. Although LLIN universal coverage is mostly achieved during these campaigns, it may not be related with net use over time. Uses given to LLINs and non-compliance of this strategy were evaluated.

**Methods:**

A knowledge, attitude and practice (KAP) cross-sectional study was conducted in Lihir Islands, PNG, 2–2.5 years after the last LLIN mass distribution campaign. Data on bed net ownership, use and maintenance behaviour was collected using a household questionnaire administered by trained community volunteers. Logistic regression models were used to identify factors associated with owning at least one LLIN and sleeping under a LLIN the previous night.

**Results:**

Among 2694 households surveyed, 27.4 % (95 % CI: 25.8–29.2) owned at least one LLIN and 8.7 % (95 % CI: 7.6–9.8) had an adequate LLIN coverage (at least one LLIN for every two people). Out of 13,595 individuals in the surveyed households, 13.6 % (95 % CI: 13.0-–4.2) reported having slept under a LLIN the preceding night. Determinants for sleeping under LLIN included living in a household with adequate LLIN coverage [adjusted OR (aOR) = 5.82 (95 % CI: 3.23–10.49)], household heads knowledge about LLINs [aOR = 16.44 (95 % CI: 8.29–32.58)], and female gender [aOR = 1.92 (95 % CI: 1.53–2.40)] (all p-values < 0.001). LLIN use decreased with older age [aOR = 0.29 (95 % CI: 0.21–0.40) for ≥ 15 year-olds, aOR = 0.38 (95 % CI: 0.27–0.55) for 5–14 year-olds] compared to < 5 year-olds (p-value < 0.001). Knowledge on the use of LLIN was good in 37.0 % of the household heads. Repurposed nets were reported serving as fishing nets (30.4 %), fruits and seedlings protection (26.6 %), covering up food (19.0 %) and bed linen (11.5 %).

**Conclusions:**

Two years after mass distribution, LLIN coverage and use in Lihir Islands is extremely low. Three yearly distribution campaigns may not suffice to maintain an acceptable LLIN coverage unless knowledge on maintenance and use is promoted trough educational campaigns.

**Supplementary Information:**

The online version contains supplementary material available at 10.1186/s12936-021-03867-z.

## Background

Universal coverage with insecticide-treated mosquito nets (ITNs) is recommended to achieve community-wide protection in malaria endemic areas [1]. In the last 3 years, more than 500 million ITNs have been distributed worldwide and undoubtedly contributed to malaria control efforts. However, coverage, maintenance and use of ITNs is heterogeneous and nets are sometimes misused or repurposed, reducing the efficiency of the overall strategy [2–4].

Long-lasting insecticidal nets (LLINs) have been massively distributed in Papua New Guinea (PNG) since 2004. Regular mass distribution campaigns were initiated in 2009 and are repeated every three years. According to a nationwide survey conducted in 2011 during the second mass distribution, 81.8 % of households retained at least one LLIN from the previous campaign [5]. Overall human malaria prevalence decreased from 15.7 % to 2009 to 4.8 % in 2011, although the reduction was more pronounced for *Plasmodium falciparum* than for *Plasmodium vivax* [6]. Moreover, a study conducted in selected sites in PNG, showed a steep decrease in malaria annual incidence: from 20 to 115 cases per 1000 population in 2010, to 1–79 cases per 1000 in 2014. This effect was attributed to LLIN mass coverage and use, rather than to the widespread use of highly effective artemisinin-based combination therapy [7]. Despite the initial success, PNG experienced a large increase of infections in the latest years, with an incidence that ramped up from 118.8 cases per 1000 inhabitants in 2015 to 184.5 cases per 1000 inhabitants in 2018 [8]. Consequently, in 2019, PNG accounted for 80 % of all malaria cases diagnosed in the Western Pacific Region [2, 9].

In view of the established efficacy of LLINs as a malaria control intervention, it is unclear why there was not more of an effect. There are several potential reasons, including insecticide resistance, vector behaviour change, incomplete campaign coverage and non-compliance to LLIN. *Anopheles* spp phenotypic resistance to pyrethroids, the insecticides commonly used in LLIN, is not yet present in PNG [10], ruling out a contribution of insecticide resistance to the changes observed in malaria epidemiology. In contrast, decreased bioefficacy of LLINs distributed between 2013 and 2019 has been described [11], as well as behavioural adaptation of local malaria vectors towards more frequent outdoor biting earlier in the evening [12, 13]. Lack of use, misuse or repurposing of LLINs may as well be related to the reduced efficacy of this particular malaria control strategy. Studies conducted in different countries have reported that repurposed nets served as fishing nets, gardening, or fencing [3, 4, 14–16]. The main reason for the lack of appropriate use and maintenance of LLIN are dislike and discomfort due to heat and perceived low mosquito density [15, 17, 18].

In this study LLIN ownership, maintenance and use in Lihir Islands were evaluated, two years after the 2016 mass distribution when 10,897 nets were issued and 97 % of individual coverage was achieved [19]. Misuse and misconception of villagers were also assessed as factors that may influence the effectiveness and sustainability of this vector control strategy over time.

## Methods

### Study setting

This study was conducted in Lihir Islands, located in the Bismarck archipelago, in New Ireland Province, PNG. New Ireland is among the PNG provinces experiencing a higher increase in malaria cases since 2015, with 426 cases per 1000 population in 2018 [20], although in previous years the province had achieved a reduction in cases comparable to other parts of the country [21, 22]. In addition, New Ireland is one of the provinces with lowest LLIN use despite high LLIN coverage according to a national indicator survey conducted in 2017 [23]. During the 2016 mass distribution campaign, 90,948 double nets were distributed by the national program in collaboration with Rotarians Against Malaria PNG (RAM), achieving a coverage ratio of 49 double-size LLINs per 100 population (97 % population coverage) [19].

Lihir islands is a group of four small islands: Aniolam (the largest, with an area of 200 km^2^), and the smaller outer islands of Malie, Masahet, and Mahur. They are characterised by a tropical rainforest climate with extremely high precipitation figures all year round. There are 8 aid posts, 1 subhealth centre and 2 health centres in the islands. A gold mine located in Aniolam is the main source of employment. Employee migration from other parts of PNG, contributes to more than one third of the population on Aniolam [24]. In 2019, Lihir had a population of approximately 27,500 inhabitants and 6000 households [25]. Households can be permanent (built of bricks, with solid material in the roof and windows with glass), traditional (built of natural materials, especially wood and grass, with open windows) or makeshift (usually made with different kinds of materials from settlement areas, such as cardboard). Malaria is one of the main health issues in Lihir islands. In 2018, 11,267 confirmed malaria cases were reported (annual incidence of 478 cases per 1000 inhabitants) with minimal variation in number of cases per month.

## Study design and procedures

A knowledge, attitude and practice (KAP) cross-sectional household survey was conducted between the 3rd of December of 2018 and the 25th of May of 2019 to assess ownership and use of LLIN distributed during 2016, before the following mass distribution campaign scheduled for late 2019.

The data collection was implemented by community volunteers, called Village Malaria Assistants (VMAs). They were selected by community leaders, and trained to implement malaria control activities at the village level, mainly awareness and education on malaria prevention, health-seeking behaviour and compliance with treatment. The VMA network in Lihir islands was established in 2018, after all VMAs were trained. A total of 40 VMAs worked in this study and were instructed to survey all households in their village or catchment area.

**Fig. 1:**
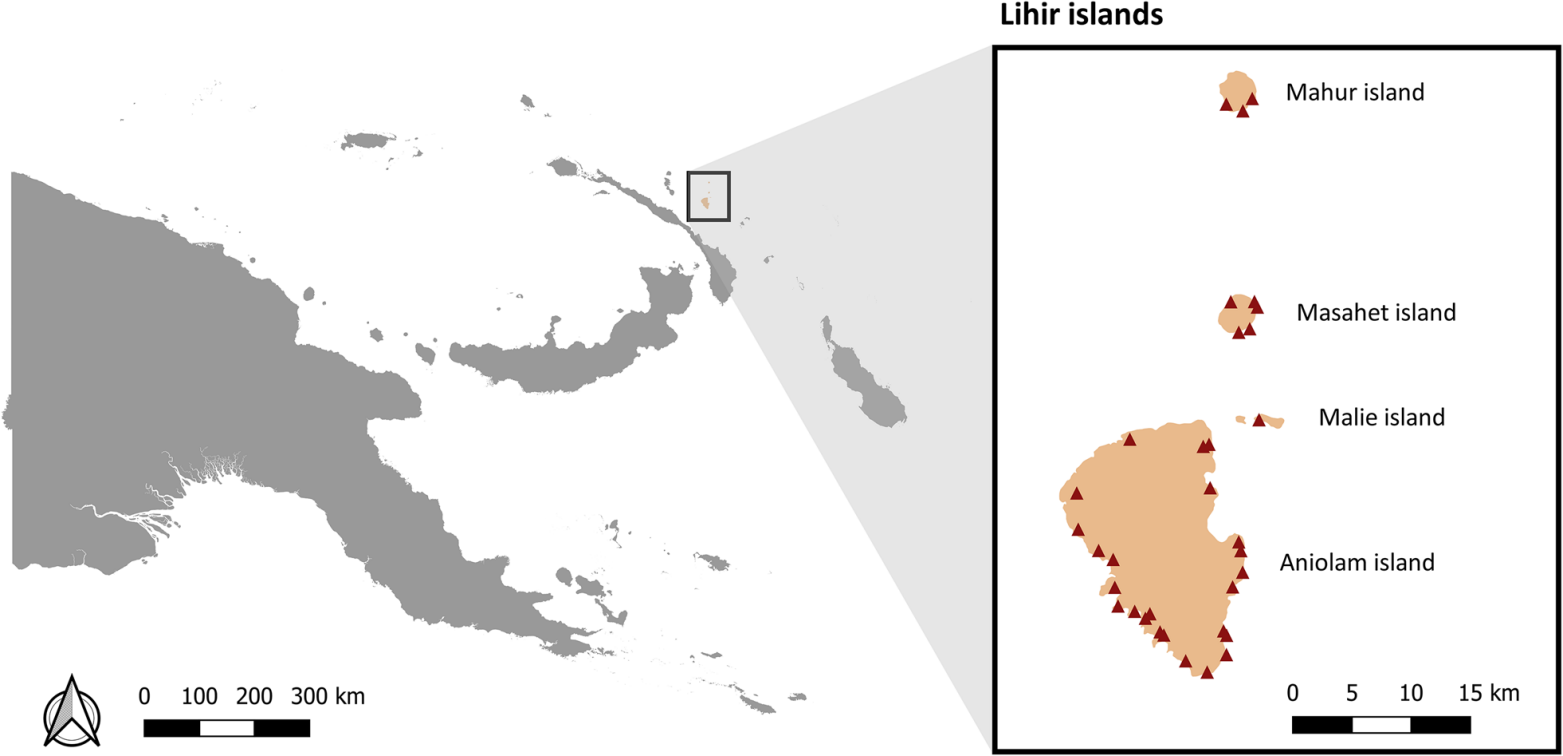
Map of the surveyed villages in the Lihir islands, New Ireland province, Papua New Guinea

The survey was conducted in a convenient sample of 33 villages out of 40 located in the four islands of the Lihir group (see Fig. 1). Although the goal was set to survey all households in Lihir islands, reaching all households was not possible due to logistical constraints; hence, households were also selected using convenience sampling. A total of 2694 households were enrolled in the survey, which represents approximately half of the households on the Lihir islands [25].

### Data collection and management

Interviews were conducted using structured questionnaires to household heads or, in its absence, to the partner or an adult permanently living in the house. A household was excluded from the study if the head of the household was not willing to participate or if no adults were available to answer the questionnaire after two separate visits.

Interviews included questions on household characteristics, demographic information of all household members (residents and long-term visitors sleeping in the same house), LLIN ownership, individual correct use of LLINs, alternative uses given to the nets, and behavioural questions related to malaria prevention. VMAs were instructed to enter the surveyed house to check the number of reported nets. Strategies to prevent malaria and alternative uses given to LLINs were asked with an open question, and household heads were prompt to list as many options as they considered relevant. After the questionnaires were collected, data were introduced into a database by two independent data clerks. See data collection tool in supplementary material, Additional file 1.

### Statistical analysis

A sample size of 379 households was estimated to detect an unknown prevalence (50 %) of having at least 1 LLIN (conservative estimate with maximum imprecision), with a margin of error of ± 5 % and a confidence level of 95 %.

Basic characteristics of participating households and individuals were summarized using descriptive analyses. Different indicators of LLIN ownership and use were calculated following the recommendations of the Roll Back Malaria Monitoring and Evaluation Reference Group [26], including: (i) proportion of households with at least one LLIN, (ii) proportion of households with at least one LLIN for every two people, (iii) proportion of population with adequate access (≥ 1 LLIN/2 individuals) to a LLIN in their household, (iv) proportion of the population that slept under a LLIN the previous night, and (v) proportion of children under five years of age who slept under a LLIN the previous night. Formulae used are shown in supplementary material, Additional file 2. Knowledge of malaria prevention tools and alternative uses given to LLIN revealed by the head of the household were reported using descriptive analyses.

Univariable and multivariable logistic regression analyses were used to identify factors associated with maintaining at least one LLIN in the household. Both models were adjusted for village as a fixed effect parameter. Similarly, univariable and multivariable mixed-effects logistic regression analyses were used to identify factors associated with sleeping under a LLIN the previous night among individuals living in a household with at least one LLIN. These two models were adjusted for household and village as random effects parameters. We have considered village as a fixed effect in the first models and random effect in the second type of models following the recommendations of Green and Tukey [27]. Analysis results are presented as odds ratios (ORs) with 95 % confidence intervals (CIs). The strength of the evidence for the different associations was calculated using likelihood-ratio tests.

Data were analysed using Stata 16 software [28]. All graphs were drawn using Stata 16 software, and a map of the surveyed villages was created using QGIS Desktop v3.16 Hannover software [29].

### Ethical considerations

The research protocol was approved by the PNG Medical Research Advisory Committee (MRAC No. 18.07). Permission to conduct this survey was also obtained from village leaders. All household heads orally consented before recording data of each household. No biological samples were collected.

## Results

### Study population

A total of 2694 households, with 13,595 individuals, were visited and enrolled in the survey. Characteristics of participating households and their residents are described in Table 1. Half of the houses (50.1 %) were permanent, 39.7 % were traditional, and 10.2 % were makeshift. The median number of people per household was 5 (interquartile range [IQR]: 3–7), and a large proportion of the household heads were male (76.6 %). Approximately half of the individuals living in the surveyed households were male (51.9 %), and 62.4 % of the population were aged 15 years and older. Most school-age children attended a local school (78.0 %), and only 22.6 % of the adults were employed.

**Table 1:** Household and study population characteristics

Variable	N (%)
Household characteristics (n = 2694)	
Type of household	
Permanent	1349 (50.1)
Traditional	1069 (39.7)
Makeshift	276 (10.2)
Number of individuals per household	
Median (IQR)	5 (3–7)
Sex of the household head	
Male	2063 (76.6)
Female	631 (23.4)
Individual chracteristics (n = 13,595)	
Gender^a^	
Male	7056 (51.9)
Female	6537 (48.1)
Age [years]^b^	
< 5	2088 (15.5)
5–14	2979 (22.1)
≥ 15	8432 (62.4)
Employed [if ≥ 15 years old]^c^	
No	5411 (77.4)
Yes	1584 (22.6)
Studying [if 5–14 years old]^d^	
No	639 (22.0)
Yes	2267 (78.0)

^a^n = 13,593 (0.01 % missing)

^b^n = 13,499 (0.7 % missing)

^c^n = 6995 (17.0 % missing)

^d^n = 2906 (2.5 % missing)

## LLIN ownership and use

Among the 2694 households responding the survey, 27.4 % (95 % CI: 25.8–29.2) owned at least one LLIN, 2–2.5 years after the mass distribution that took place in the islands. A total of 416 households had one LLIN, 117 had two LLINs, 183 had three LLINs, and 23 had four or more LLINs. Only 8.7 % (95 % CI: 7.6–9.8) of households had at least one LLIN for every two individuals (adequate household coverage). Similarly, the percentage of people with adequate access to a LLIN within their household was estimated to be 6.7 % (95 % CI: 6.2–7.1).

**Fig. 2:**
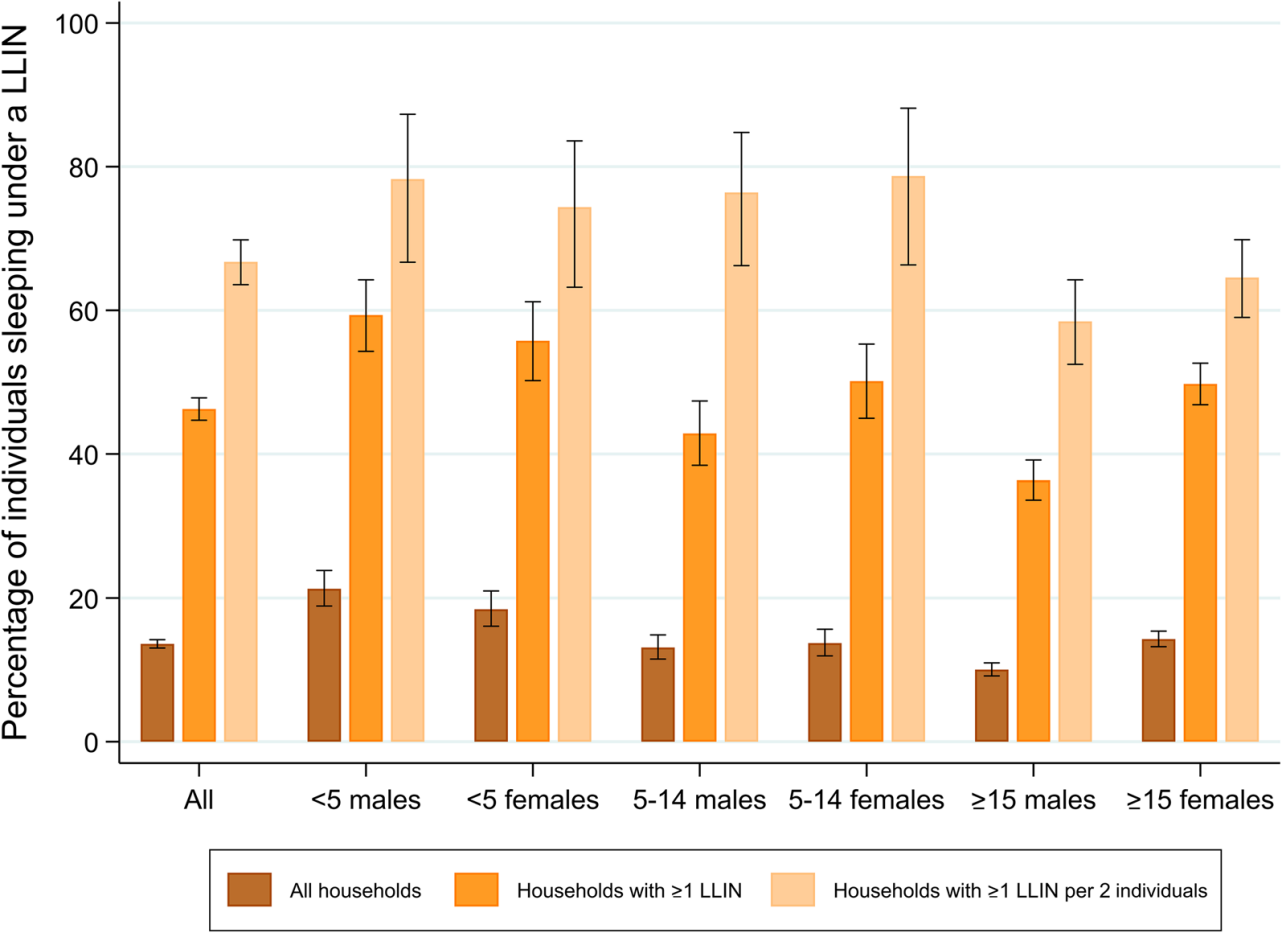
Percentage of individuals reported to sleep under a LLIN the previous night by sex and age groups, among (1) all households, (2) households that have at least 1 LLIN, and (3) households with at least 1 LLIN per 2 individuals

**Fig. 3:**
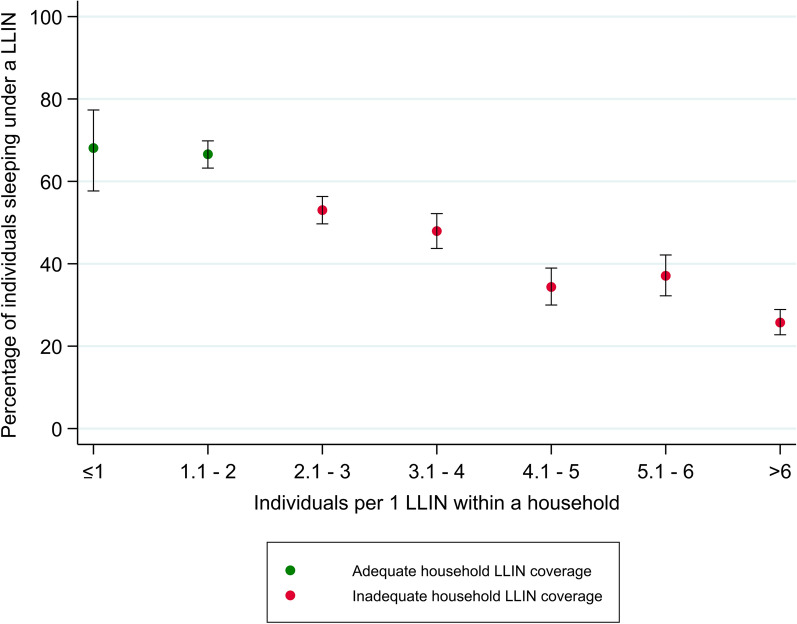
Percentage of individuals reported to sleep under a LLIN the previous night by the ratio individuals:LLIN of the household, among the 739 households with at least 1 LLIN

Regarding LLIN use, a total of 1851 individuals [13.6 % (95 % CI: 13.0–14.2)] reported to have slept under a LLIN the previous night. Among individuals living in a household with at least one LLIN, a 46.3 % (95 % CI: 44.7–47.8) slept under a LLIN the previous night. Among individuals living in a household with adequate LLIN coverage, a 66.7 % (95 % CI: 63.6–69.8) did so. A detailed breakdown of individuals using LLIN by age and sex according to household LLIN coverage is shown in Fig. 2. Among the key population of children under five years of age, 19.9 % (95 % CI: 18.2–21.7) reported to have slept under a LLIN the previous night. The percentage of individuals reporting to have slept under a LLIN increased with LLIN availability within a household (Fig. 3).

## Determinants of owning at least one LLIN

Factors associated with LLIN ownership are shown in Table 2. In the multivariable analysis, owning at least one LLIN was associated with having at least one resident aged < 5 years-old [adjusted OR (aOR) = 1.55 (95 % CI: 1.17–2.06), p-value = 0.002], having at least one resident being an adult woman [aOR = 1.82 (95 % CI: 1.04–3.16), p-value = 0.029], and with the head of the household knowing that sleeping under a LLIN prevents malaria [aOR = 30.32 (95 % CI: 21.25–43.27), p-value < 0.001].

**Table. 2:** Factors associated with household ownership of at least one LLIN (n = 2694 households)

Variable	Total households	Owning ≥ 1 LLIN (%)	Univariable aOR (95 %CI)	p-value	Multivariable aOR (95 %CI)	p-value
Type of household						
Permanent	1349	360 (26.7)	1	0.099	1	0.405
Traditional	1069	289 (27.0)	0.91 (0.74–1.13)		0.90 (0.67–1.21)	
Makeshift	276	90 (32.6)	1.34 (0.97–1.85)		1.21 (0.79–1.86)	
Number of individuals per household						
Median (IQR)	5 (3–7)	5 (4–7)	1.07 (1.03–1.12)	0.001	1.02 (0.96–1.09)	0.529
Household head knows that sleeping under a LLIN prevents malaria						
No	1697	117 (10.4)	1	< 0.001	1	< 0.001
Yes	997	562 (56.4)	24.08 (17.96–32.30)		30.32 (21.25–43.27)	
Gender of the household head						
Male	2063	555 (26.9)	1	0.690	1	0.908
Female	631	184 (29.2)	1.05(0.83–1.32)		0.98 (0.71–1.35)	
At least 1 resident aged < 5 years^a^						
No	1320	288 (21.8)	1	< 0.001	1	0.002
Yes	1347	443 (32.9)	1.70 (1.39–2.06)		1.55 (1.17–2.06)	
At least 1 resident an adult woman [> 15 years old]						
No	241	38 (15.8)	1	< 0.001	1	0.029
Yes	2453	701 (28.6)	2.35 (1.56–3.53)		1.82 (1.04–3.16)	
At least 1 resident employed [> 15 years old]^b^						
No	1129	268 (23.7)	1	0.022	1	0.579
Yes	1122	340 (30.3)	1.29 (1.04–1.61)		1.08 (0.82–1.43)	
At least 1 resident studying [5–14 years old]^c^						
No	1342	334 (24.9)	1	0.108	1	0.167
Yes	1315	391 (29.7)	1.17 (0.97–1.43)		0.81 (0.61–1.09)	

^a^n = 2667 (1.0 % missing)

^b^n = 2251 (16.4 % missing)

^c^n = 2657 (1.4 % missing)

## Determinants of sleeping under LLIN

Out of 4,002 individuals that lived in a household with at least one LLIN, 46.3 % (95 % CI: 44.7–47.8) reported sleeping under a LLIN the preceding night. Table 3 shows the factors associated with LLIN use. In the multivariable analysis, sleeping under a LLIN the previous night was associated with living in a household with adequate LLIN coverage [adjusted OR (aOR) = 5.82 (95 % CI: 3.23–10.49)], head of household knowing that sleeping under a LLIN prevents from malaria [aOR = 16.44 (95 % CI: 8.29–32.58)], being female [aOR = 1.92 (95 % CI: 1.53–2.40)] and decreasing age [aOR = 0.38 (95 % CI: 0.27–0.55) for 5–14 year-olds, and aOR = 0.29 (95 % CI: 0.21–0.40) for ≥ 15 year-olds, compared both to < 5 year-olds] (all p-values < 0.001).

**Table 3:** Factors associated with sleeping under a LLIN among individuals residing in households owning at least one LLIN (n = 4002 individuals)

Variable	Total individuals	Slept under a LLIN the previous night (%)	Univariable aOR (95 %CI)	p-value	Multivariable aOR (95 %CI)	p-value
Gender						
Male	2093	879 (42.0)	1	< 0.001	1	< 0.001
Female	1909	972 (50.9)	1.99 (1.64–2.41)		1.92 (1.53–2.40)	
Age [years]^a^						
< 5	721	416 (57.7)	1	< 0.001	1	< 0.001
5–14	867	399 (46.0)	0.37 (0.27–0.51)		0.38 (0.27–0.55)	
≥ 15	2373	1021 (43.0)	0.32 (0.24–0.41)		0.29 (0.21–0.40)	
Type of household						
Permanent	2168	917 (42.3)	1	0.021	1	0.200
Traditional	1402	738 (52.6)	2.08 (1.26–3.42)		1.44 (0.82–2.52)	
Makeshift	432	196 (45.4)	0.99 (0.47–2.06)		0.66 (0.29–1.53)	
Household LLIN coverage						
Inadequate (< 1 LLIN per 2 individuals)	3097	1247 (40.3)	1	< 0.001	1	< 0.001
Adequate (≥ 1 LLIN per 2 individuals)	905	604 (66.7)	6.61 (3.99–10.96)		5.82 (3.23–10.49)	
Household head knows that sleeping under a LLIN prevents malaria						
No	982	211 (21.5)	1	< 0.001	1	< 0.001
Yes	3020	1640 (54.3)	20.84 (11.48–37.85)		16.44 (8.29–32.58)	
Gender of the household head						
Male	3081	1443 (46.8)	1	0.696	1	0.252
Female	921	408 (44.3)	0.89 (0.52–1.53)		0.68 (0.36–1.28)	
At least 1 resident employed [> 15 years old]^b^						
No	1147	623 (54.3)	1	0.028	1	0.082
Yes	2044	924 (45.2)	0.54 (0.32–0.90)		0.62 (0.37–1.04)	
At least 1 resident studying [5–14 years old]^c^						
No	1399	695 (49.7)	1	0.150	1	0.670
Yes	2528	1124 (44.5)	0.70 (0.45–1.11)		0.89 (0.53–1.49)	

^a^n = 3961 (1.0 % missing)

^b^n = 3191 (20.3 %missing)

^c^n = 3927 (1.9 %missing)

## Knowledge of malaria prevention tools

**Fig. 4:**
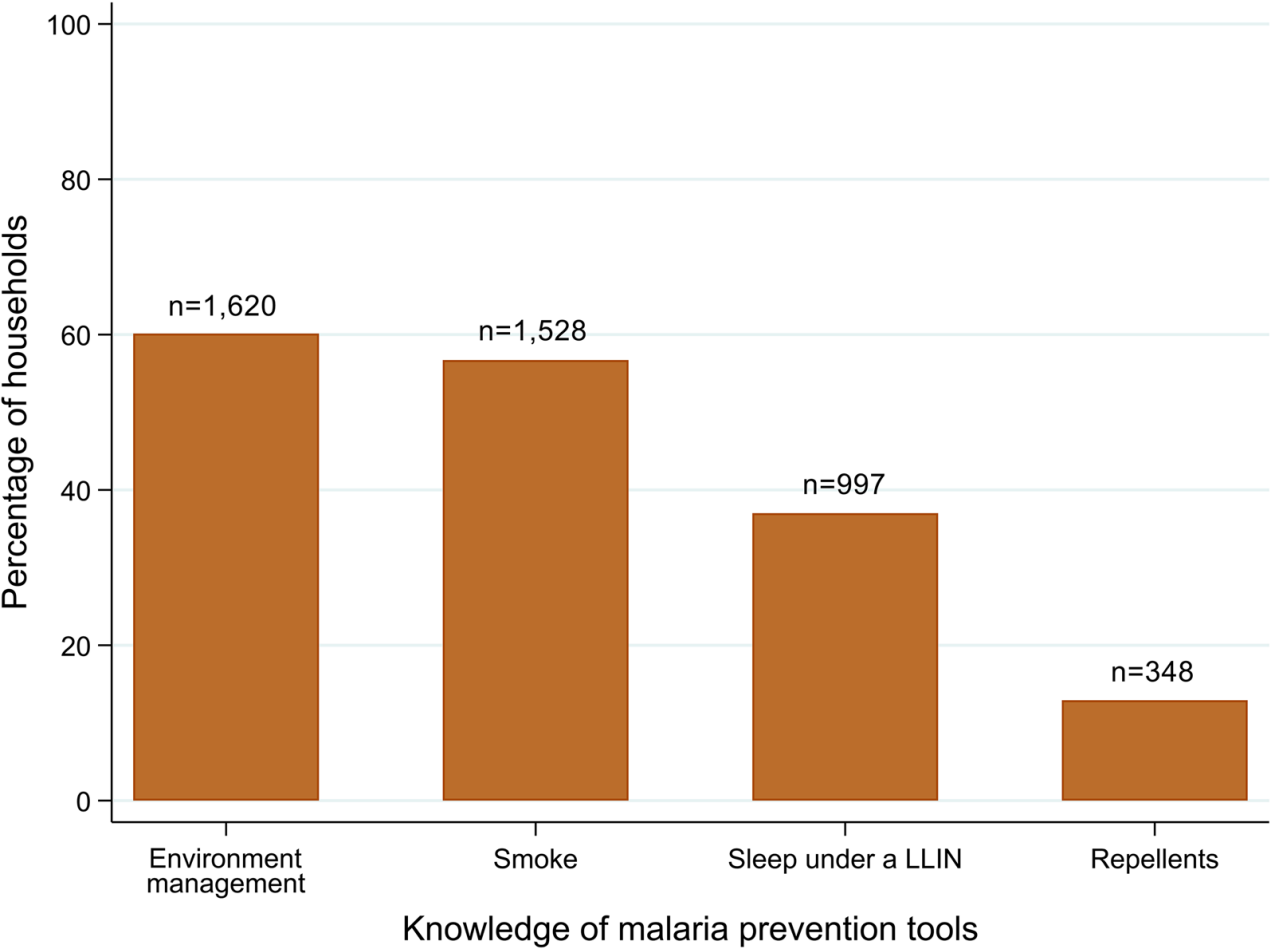
Knowledge of malaria prevention tools reported by the household head (n = 2694 households)

When the head of the household was asked about strategies to prevent malaria, 37.0 % (997/2694) responded that sleeping under a LLIN was effective to prevent malaria. Most of them (1620; 60.1 %) answered that strategies of environmental management are effective, including cleaning the house and removing water from gardens, and/or cleaning villages. Another frequent answer (1528; 56.7 %) was that creating smoke by burning bush material was useful to prevent malaria, while the use of mosquito repellents as a malaria prevention tool was pointed out by only 12.9 % of the household heads. Figure 4 shows the knowledge on malaria prevention tools by household heads.

## Alternative uses of LLIN

**Fig. 5:**
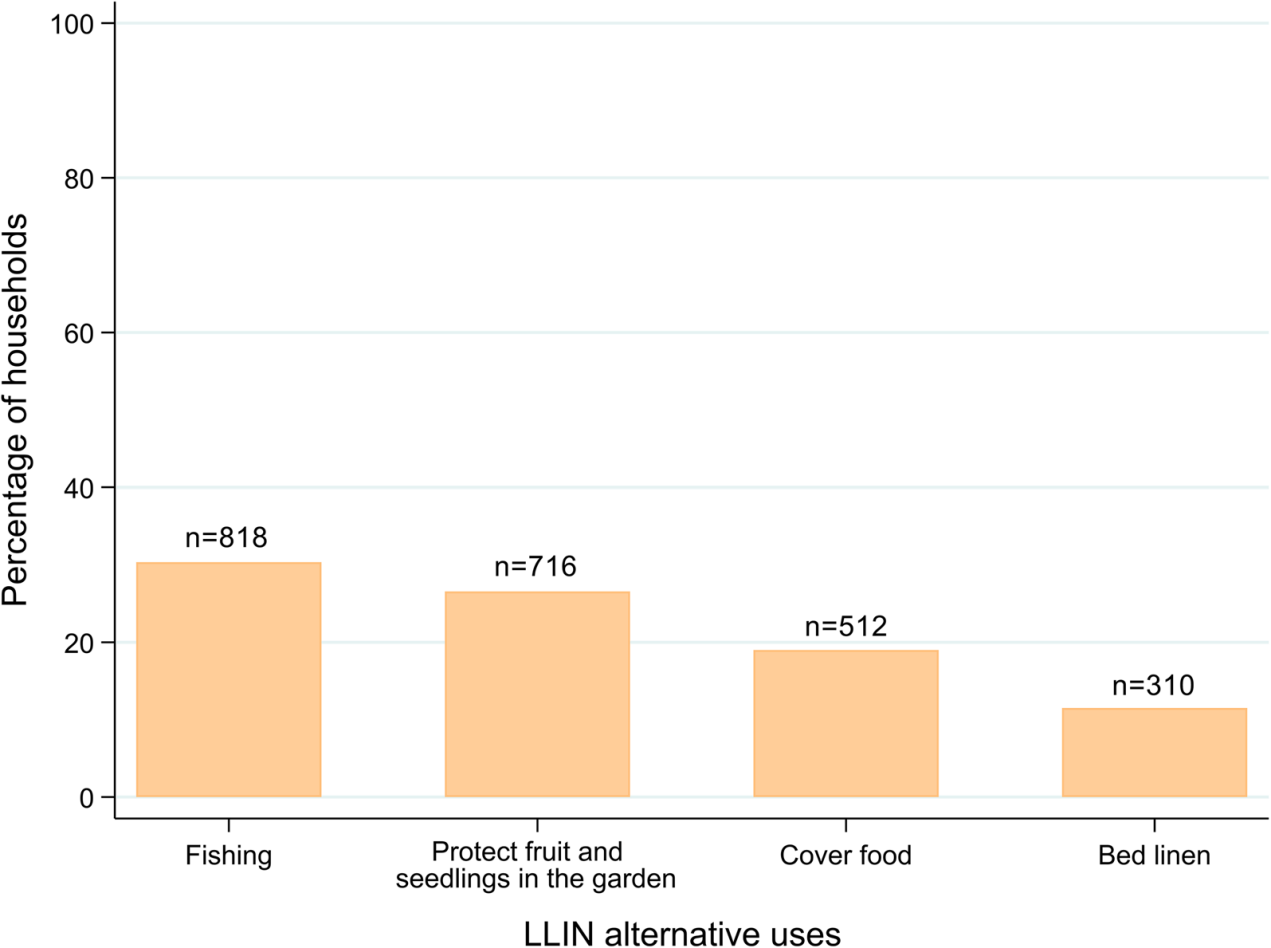
Alternative uses of LLIN reported by the household head (n = 2694 households)

When asked about use of LLINs, a total of 1170 (43.4 %) household heads reported to have used LLINs only for the purpose of sleeping under. Many gave alternative uses (either misuse or repurposing) to the LLINs: 937 (34.8 %) households provided one alternative use, 401 (14.9 %) households provided two alternative uses, 127 (4.7 %) provided three, and 59 (2.2 %) provided four or more. The most common alternative use for LLINs was fishing (818; 30.4 %), followed by protecting fruits and seedlings in gardens (716, 26.6 %). Other uses given to the LLINs included covering food in the house (512, 19.0 %) and using them as bed linen (310, 11.5 %). Figure 5 shows the alternative uses given to the LLINs.

## Discussion

This study shows a very large reduction over time on the adequate LLIN coverage in Lihir Islands, decreasing from 97 % of individuals having access to LLIN after 2016 mass distribution [19] to less than 7 % in 2–2.5 years (one year prior to the next mass distribution campaign). This reduction in LLIN coverage is rather striking, and significantly different to the relatively high bed net coverage maintained in previous distributions campaigns in the country. As an example, a 2011 study in selected sites in PNG showed that 88.8 % of people still had access to LLIN in villages where the distribution had been conducted during the 2 years preceding the survey, whereas coverage decreased to 67.6 % in those villages where the distribution had been done more than 2 years before the survey [5]. Lack of LLIN maintenance is a common issue in other tropical areas: in Uganda, LLIN population coverage decreased from 65 to 18 % three years after distribution [30]; and in Tanzania, households with at least one LLIN for every two people were below 30 % two years after LLIN distribution [31]. Accordingly, only less than 14 % of the total population surveyed slept under LLIN the previous night; and adequate LLIN coverage was strongly associated with LLIN use as previously reported [5, 32], confirming the importance to achieve high coverage and maintenance to sustain LLIN use.

Interestingly, while adequate LLIN coverage in PNG has improved over time, especially in the islands’ region where Lihir Islands are located (increasing from 46 % to 2009 to 82 % in 2011), use of LLIN still remained poor: 25 % of individuals in this region reported sleeping under LLIN in 2009, which increased to only 40 % in 2011 despite the substantial improve in LLIN coverage [33]. In fact, the villages in the PNG islands’ region are those with better LLIN coverage but worse LLIN use [23]. Hence, achieving adequate LLIN coverage during mass distribution is clearly not sufficient to ensure their use. A qualitative study in PNG found that many environmental, human and nets factors could be linked to the impediments of the adequate use of nets; however, the most important factor that reduced the use was a lack of fear of malaria infection [34]. Promoting LLIN maintenance over time is also key to enhance their use and impact on malaria transmission. Mass LLIN distribution campaigns in PNG, similar as to many other countries, achieve a high coverage in the minimum time possible using strategies that are proven to work such as use of coupons or training of distributors [35]. Including additional strategies to target issues affecting long-term coverage could enhance maintenance and use of LLIN until the following mass distribution.

Households with at least one child under 5 years-old and households with at least one adult woman had a higher odds of owning at least one LLIN similar to other studies in PNG [33]. The scale up of antenatal services in the country, where pregnant women are targeted for prevention strategies and receive LLIN [36], has likely contributed to increase LLIN ownership in their households and probably enhanced maintenance. Antenatal services provide awareness on the importance of pregnant women and children below 5 years old to sleep under LLIN to prevent malaria. Interestingly, gender and age were associated with LLIN use, with women being more likely to sleep under LLIN as well as the younger members of the household.

However, the key factor for LLIN ownership and use was the head of the household’s knowledge about LLIN preventing from malaria infection. Only 37 % of the heads of the households reported that sleeping under a LLIN was effective to prevent malaria. These results suggest that education campaigns on malaria prevention tools targeting the heads of the households could further promote LLIN maintenance and use, as shown in previous studies [37]. Such education campaigns could be included as part of the mass LLIN distribution strategy, which could also look for the support of community leaders, pastors and other influential community members to deliver key awareness messages. In Lihir islands, the VMAs deployed an extensively education campaign at village and hamlet level during the LLIN distribution in 2019, targeting the heads of the households and involving community and church leaders. This intensive education campaign had not been conducted previously and it may have improved LLIN coverage, use and maintenance.

In addition, although an association between a household having at least one resident attending school and increased odds of owning one LLIN or sleeping under a LLIN was not shown, half of the households had at least one child between 5- and 14- years old attending school. Consequently, there is also a big opportunity to promote LLIN maintenance and use through frequent education campaigns in schools. In Tanzania, for example, nets were provided annually to children attending primary and secondary school, which resulted in high level of maintenance (50–75 % of nets given) over the first four years of distribution, even in the absence of a mass distribution campaign [38].

On the other hand, addressing alternative uses and repurposing of LLIN could also enhance maintenance and use. In this study, half of the surveyed households admitted using LLIN for alternative purposes. The study was unable to determine if LLIN used for alternative purposes were those provided in the distribution campaign in 2016 or those remaining from the 2013 campaign. However, since most households did not retain a single LLIN, it is likely that most nets used for other purposes were those given in 2016. The most common alternative use given to LLIN was fishing, which could be related to the low LLIN maintenance and use observed in the PNG islands’ region, among other factors. Another common alternative use was to protect seedlings and food. These common misuses have been also described in sub-Saharan Africa [4, 12–14] and all relate to basic needs like ensuring food supply. LLIN distribution using mass campaigns are proven to increase LLIN ownership [39–41]; however, especially when health education might not be sufficient to reduce alternative uses of LLIN when other basic needs are involved, some creative solutions could be used during mass LLIN distribution campaigns, such as facilitating access of target communities to suitable and without insecticide materials for fishing and gardening. In addition, ensuring high LLIN bioefficacy and teaching communities about the impact of LLIN in reducing mosquito population could further motivate communities to better maintain LLIN.

This study used a community approach that allowed a massive outreach to the Lihirian population but also had some limitations. Although the goal of VMAs was to survey their entire village or assigned part of a village, full coverage of all villages was not possible due to logistical constraints. Hence, it could be subjected to selection bias. However, all statistical models were adjusted for village in order to minimize bias arising from the non-representativeness of a few villages. In addition, our sample size was very large and spatio-geographically and demographically representative. Because data collection was conducted by the VMAs who are living in the same village, sensitive questions such as socioeconomic factors or enquiring about pregnancy status were avoided. These two pieces of information could have given important insight, such as LLIN use during pregnancy or the role of socioeconomic status contributing to repurposing of LLIN. It was observed that, while less than 7 % of individuals had adequate access to LLIN, close to 14 % were reported to sleep under them, which could be due to more than two individuals sharing a double net (as commonly seen for young children) and due to the social desirability bias inherent in self-reported measures. Finally, in order to maximize quality of the results, VMAs received an intensive training and close supervision, and data were carefully reviewed to recode impossible values and minimize missing values.

## Conclusions

Although mass LLIN distribution campaigns are a proven health intervention to promote LLIN ownership and use, and reduce the malaria burden, distribution every three years does not seem to be sufficient to maintain an adequate LLIN coverage in the Lihir islands, PNG. Knowledge on malaria prevention tools by household heads is a determinant factor for retention and use of LLINs, as well as strategies targeting risk groups like pregnant women and children below 5 years of age. Thus, it is extremely important to ensure education of local communities in how to use and maintain the LLINs distributed to sustain the achieved high coverage during mass distribution for as long as possible and maximize impact for malaria control. Community approaches to gather information through trained community volunteers are useful to understand and deploy public health strategies. Lack of maintenance and use of LLIN, together with reduced LLIN bioefficacy and changes in mosquito biting behaviour, might altogether explain the recent increase in malaria cases observed in PNG.

## Supplementary Information

**Additional file 1**: Data collection tool used by the village malaria assistants (VMA) tocollect information for this survey.

**Additional file 2**: Definitions of the LLIN ownership and use indicators, used for thedata analysis.

## Data Availability

The datasets used and analysed during the current study are available from the corresponding author on reasonable request.

## Abbreviations

aOR: Adjusted odds ratio
CI: Confidence interval
IQR: Interquartile range
ITN: Insecticide-treated net
KAP: Knowledge, attitude and practice
LLIN: Long-lasting insecticidal nets
OR: Odds ratio
PNG: Papua New Guinea
RAM: Rotarians Against Malaria
VMA: Village malaria assistant
