# Supplementary material for: Coverage, determinants of use and repurposing of long-lasting insecticidal nets two years after a mass distribution in Lihir Islands, Papua New Guinea: a cross-sectional study
Source: Malar J. 2021 Aug 4;20:336. doi: 10.1186/s12936-021-03867-z (PMC8336363; doi:10.1186/s12936-021-03867-z)
Supplement: Supplementary file 1 — Additional file 1: Data collection tool used by the village malaria assistants (VMA) tocollect information for this survey. [file 12936_2021_3867_MOESM1_ESM.docx]

**ADDITIONAL FILE 1. DATA COLLECTION TOOL.**

**
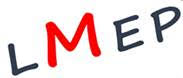
**

**HOUSEHOLD BED NET INVENTORY**

**Lihir Malaria Elimination Program Charitable Trust**

**Ward No________ Village ____________ Hamlet __________**

**Head of household:**

**Name of interviewer: ______________**

**Date: ___/___/_____**

1. What is the type of house? Permanent Traditional Makeshift

2. How many rooms in the house? 1 2 3 4+

3. Do you have mosquito nets in this house? YES NO

4. How many mosquito nets are in the house? __________

5. How many people living in this house? ____________

List of people living in the house

| Number | Sex | Age | | | Slept under bed net last night? | | Employed | Unemployed | Student | Not yet in school |
| --- | --- | --- | --- | --- | --- | --- | --- | --- | --- | --- |
|  | M/F | <5 y | 5-14 y | 15+ y | YES | NO |  |  |  |  |
| 1. Household head |  |  |  |  |  |  |  |  |  |  |
| 2. |  |  |  |  |  |  |  |  |  |  |
| 3. |  |  |  |  |  |  |  |  |  |  |
| 4. |  |  |  |  |  |  |  |  |  |  |
| 5. |  |  |  |  |  |  |  |  |  |  |
| 6. |  |  |  |  |  |  |  |  |  |  |
| 7. |  |  |  |  |  |  |  |  |  |  |
| 8. |  |  |  |  |  |  |  |  |  |  |
| 9. |  |  |  |  |  |  |  |  |  |  |
| 10. |  |  |  |  |  |  |  |  |  |  |

| 6. What do you do to avoid malaria?  _____________________________________  ____________________________________  Classify:   \| Sleep under LLIN \| 1 \| \| --- \| --- \| \| Smoke/herbs from fire \| 2 \| \| Drink malaria medicine \| 3 \| \| Environmental manipulation \| 4 \| \| Insecticide house/body \| 5 \| \| Others \| 8 \| | 7. Other uses of bed nets in this house  _________________________________________________________  Classify:   \| Protect ripe fruits \| 1 \| \| --- \| --- \| \| Cover food \| 2 \| \| Cover seedlings \| 3 \| \| Fishing in river/see \| 4 \| \| Cover as bedlinen \| 5 \| \| Others \| 8 \| \| No other uses (only sleep under) \| 0 \| |
| --- | --- | --- | --- | --- | --- | --- | --- | --- | --- | --- | --- | --- | --- | --- | --- | --- | --- | --- | --- | --- | --- | --- | --- | --- | --- | --- | --- |
