# Supplementary material for: Coverage, determinants of use and repurposing of long-lasting insecticidal nets two years after a mass distribution in Lihir Islands, Papua New Guinea: a cross-sectional study
Source: Malar J. 2021 Aug 4;20:336. doi: 10.1186/s12936-021-03867-z (PMC8336363; doi:10.1186/s12936-021-03867-z)
Supplement: Supplementary file 2 — Additional file 2: Definitions of the LLIN ownership and use indicators, used for thedata analysis. [file 12936_2021_3867_MOESM2_ESM.docx]

**ADDITIONAL FILE 2- SUPPLEMENTARY MATERIAL**

Definitions of the LLIN ownership and use indicators

| INDICATOR | DEFINITION | |  |
| --- | --- | --- | --- |
| Proportion of households with at least one LLIN | | $\frac{Number of households surveyed with \geq1 LLIN}{Total number of households surveyed}\cdot100$ | |
| Proportion of households with at least one LLIN for every two people | | $\frac{Number of households with \geq1 LLIN for every 2 people}{Total number of households surveyed}\cdot100$ | |
| Proportion of population with adequate access to a LLIN in their household | | $\frac{Number of individuals who could sleep under a LLIN if each LLIN in the household were used by 2 people}{Total number of individuals in the surveyed households}\cdot100$ | |
| Proportion of the population that slept under a LLIN the previous night | | $\frac{Number of individuals who slept under a LLIN the previous night}{Total number of individuals in the surveyed households}\cdot100$ | |
| Proportion of children under five years old who slept under a LLIN the previous night | | $\frac{Number of children under 5 years old who slept under a LLIN the previous night}{Total number of children under 5 years old in the surveyed households}\cdot100$ | |
